# The 2010 American college of rheumatology fibromyalgia survey diagnostic criteria and symptom severity scale is a valid and reliable tool in a French speaking fibromyalgia cohort

**DOI:** 10.1186/1471-2474-13-179

**Published:** 2012-09-20

**Authors:** Mary-Ann Fitzcharles, Peter A Ste-Marie, Pantelis Panopalis, Henri Ménard, Yoram Shir, Fred Wolfe

**Affiliations:** 1 Division of Rheumatology, McGill University, Montreal, Canada; 2 Alan Edwards Pain, Management Unit, Montreal, Canada; 3 Faculty of Law, Université de Montréal, Montreal, Canada; 4 National Data Bank for Rheumatic Diseases and University of Kansas School of Medicine, Wichita, KS, USA; 5 Montreal General Hospital, McGill University Health Centre, 1650 Cedar ave, Montreal, Quebec, H3G 1A4, Canada

**Keywords:** Fibromyalgia, Criteria, Severity scale, French

## Abstract

**Background:**

Fibromyalgia (FM) is a pain condition with associated symptoms contributing to distress. The Fibromyalgia Survey Diagnostic Criteria and Severity Scale (FSDC) is a patient-administered questionnaire assessing diagnosis and symptom severity. Locations of body pain measured by the Widespread Pain Index (WPI), and the Symptom Severity scale (SS) measuring fatigue, unrefreshing sleep, cognitive and somatic complaints provide a score (0–31), measuring a composite of polysymptomatic distress. The reliability and validity of the translated French version of the FSDC was evaluated.

**Methods:**

The French FSDC was administered twice to 73 FM patients, and was correlated with measures of symptom status including: Fibromyalgia Impact Questionnaire (FIQ), Health Assessment Questionnaire (HAQ), McGill Pain Questionnaire (MPQ), and a visual analogue scale (VAS) for global severity and pain. Test-retest reliability, internal consistency, and construct validity were evaluated.

**Results:**

Test-retest reliability was between .600 and .888 for the 25 single items of the FSDC, and .912 for the total FSDC, with all correlations significant (p < 0.0001). There was good internal consistency measured by Cronbach’s alpha (.846 for FSDC assessment 1, and .867 for FSDC assessment 2). Construct validity showed significant correlations between the FSDC and FIQ 0.670, HAQ 0.413, MPQ 0.562, global VAS 0.591, and pain VAS 0.663 (all p<0.001).

**Conclusions:**

The French FSDC is a valid instrument in French FM patients with reliability and construct validity. It is easily completed, simple to score, and has the potential to become the standard for measurement of polysymptomatic distress in FM.

## Background

The cornerstone symptom of fibromyalgia (FM) is chronic widespread pain, with presence of associated symptoms of fatigue, unrefreshing sleep, cognitive dysfunction, and a variety of somatic symptoms that may be present to variable degree in an individual patient [1,2]. It is this combination of symptoms that constitutes the overall suffering or distress of patients with chronic pain conditions or FM. To date, there is no consensus regarding the ideal measurement to assess symptom severity in FM, or to follow patients regarding outcome or change in symptoms.

The only questionnaire that is disease specific for FM and addresses a number of domains for this condition is the Fibromyalgia Impact Questionnaire (FIQ), although with criticism that it does not fully address all aspects of FM, may not be sufficiently sensitive to change, and is complex to score [3,4]. Other assessments for FM patients have focused on individual symptoms such as pain (i.e. visual analogue scale [VAS], McGill Pain Questionnaire [MPQ], Pain Disability Index [PDI]), fatigue (i.e. Multidisciplinary Fatigue Inventory), sleep disturbance and mood (i.e. Hospital Anxiety and Depression scale [HADS]), but all used almost entirely in the research setting [3,5-9]. There is therefore a need for an assessment tool that may be easily applied in both the clinical and research setting, is simple to score, and gives meaningful information about the overall status of a patient with FM.

The Fibromyalgia Survey Diagnostic Criteria and Severity Scale (FSDC) is a patient administered questionnaire that captures the key symptoms of FM, assesses symptom severity, and has the potential to be a useful instrument that can be applied in clinical care [10,11]. However, the diagnosis requires a medical examination to rule out other somatic diseases that could sufficiently explain the symptoms. The FSDC has been validated in English speaking patients with FM as well as Japanese patients with and without FM, and assesses the locations of body pain as measured by the Widespread Pain Index (WPI), and the associated symptom severity (SS) of fatigue, unrefreshing sleep, cognitive complaints, and somatic symptoms [10,12]. The sum of the WPI and the SS provides a score (0–31) which is a measurement of symptom severity representing polysymptomatic distress. In this current study we report on the validity and reliability of a French translated version of the FSDC in a tertiary clinic population of FM patients.

## Methods

### Study design

Eighty francophone patients with FM were invited to participate in this study, 7 of whom did not fully complete the questionnaires. Therefore, the study sample comprised 73 patients with an existing diagnosis of FM, usually made by a family physician and confirmed as a clinical diagnosis by a rheumatologist. Fifty-nine consecutive patients are current participants in a prospective cohort study in a multidisciplinary pain centre. The remaining 14 participants, members of a patient support group, were assessed by the study rheumatologist but were unable to participate in the cohort study due to distance from the study centre, and did not differ from the cohort study participants. All patients provided informed consent and ethical approval was obtained from the institutional review board of the Montreal General Hospital.

### Study procedure

At the time of the first assessment the following information was obtained: demographic and disease related information, questionnaires assessing symptoms and function associated with FM, and the FSDC. All questionnaires were validated French language versions with the exception of the FSDC. The patients were asked to complete the FSDC on a second occasion, within 7 days, and the questionnaire was returned by mail. If not returned, the study participant was prompted with a telephone call, and if the questionnaire was not returned within 2 weeks, the participant was counted as a study withdrawal.

### Assessment tools

#### Symptoms and physical functioning

Patients completed the FIQ, a validated disease specific instrument assessing the health status of patients with FM [3]. The FIQ is commonly used as a primary endpoint in clinical trials of FM. It consists of 19 subscales assessing physical function, number of days feeling bad, work missed, job ability, pain, fatigue, morning tiredness, stiffness, anxiety and depression. The maximum score for the FIQ is 100, with higher values indicating greater severity. Functional status was also measured with the Health Assessment Questionnaire disability index (HAQ) [13]. Fatigue and patient global assessment of disease severity were assessed by a 10 cm VAS for each parameter.

#### Pain assessment

Pain was assessed by a number of parameters. Pain intensity was measured by a 10 cm VAS. Pain quality was measured by means of the MPQ, a validated questionnaire comprising 78 descriptor words arranged into 20 subgroups and measuring the sensory, affective, evaluative, and miscellaneous components of pain [6]. Patients are asked to circle the word from each subgroup which most accurately describes their pain. The total MPQ intensity score is calculated by summing the total number of words weighted by each word’s rank order within its subcategory [6]. Pain-related interference with role functioning was measured with the Pain Disability Index (PDI), a validated generic measure of function applicable to varied painful conditions with measures in 7 areas (occupational, home/family, recreational, social, sexual, activities of daily living and life support), all rated on 11-point Likert type scale (0, no disability, 10, complete disability). The maximum score is 70, with higher score indicating greater severity [7,14]. A manikin of the body with a front and back view was shaded to indicate location of pain. This was scored according to the quantitative method recommended by Staud and colleagues that measures a total of 50 areas (26 back and 24 front) [15].

#### Fibromyalgia Survey Diagnostic Criteria (FSDC)

The FSDC is a patient administered questionnaire comprising 3 sections [10]. The first section contains 3 questions on symptoms of fatigue, cognitive problems, and unrefreshing sleep during the past week, each of which is scored by a Likert format from 0 (no problem) to 3 (severe: continuous, life-disturbing problems). The scores are summed with a maximum score of 9. The second section comprises 3 questions with a positive or negative response for the following somatic complaints occurring during the past 6 months, abdominal pain or cramps, depression, and headache, with a maximum score of 3. The sum of section 1 and 2 provides a Symptom Severity Score (SS), with a range from 0–12. The third section is a measurement of the Widespread Pain Index (WPI) and is completed by identifying body areas where pain or tenderness was felt during the previous 7 days, with a total of 19 body areas identified as follows: shoulder girdle (left and right), upper arm (left and right), lower arm (left and right), hip (left and right), upper leg(left and right), lower leg (left and right), jaw (left and right), chest, abdomen, upper back, lower back, and neck. The maximum score for the WPI section is 19. The fibromyalgianess scale (FS) is defined as the sum of the (0–19) WPI and the 6-item (0–12) SS scale. It has a range of 0–31.

### Translation of the FSDC

The translation protocol used to translate the FSDC is based on methodology that has been previously published [16,17] and applied to obtain Canadian French versions of evaluation instruments [18]. With the permission of the authors, the FSDC was translated into French as follows. We obtained two initial forward translations of the instrument into French by two bilingual individuals, one with health-related experience and the other from the lay public, whose first language is French. The two forward translations were then back-translated into English by two bilingual individuals, one with health-related experience and the other from the lay public, whose mother tongue is English. The original version, the two French forward translations, and the two English back translations were reviewed by a committee composed of two investigators, one whose mother tongue is French and the other English, and one of the individuals involved in the back translation. The committee members worked by consensus to finalize a single French version of the FSDC, ensuring the conceptual and linguistic equivalence of the two versions.

The experimental version of the instrument was tested on a sample of 5 bilingual patients to ensure clarity and comprehension. They completed both the French and English versions of the instrument, in random order, and indicated if they found any instructions or items difficult or ambiguous. An investigator was present for cognitive debriefing. Any items for which difficulties were encountered in the French translation, but not in the original version, were re-worded by the investigators. We assessed the test-re-test reliability of the French Canadian version by administering the instrument to a group of French-speaking patients twice within a 1 week period.

### Statistical analysis

Test-retest reliability of the FSDC was assessed with Pearson’s correlation coefficients. To assess internal reliability, an analysis of internal consistency using Cronbach’s coefficient α was performed with measurement of .9 < α ≥ .8 considered as representing good internal consistency. To test construct validity we hypothesized a strong to moderate relationship between the FSDC and other measures of symptom severity in FM including the FIQ and HAQ, and symptoms of fatigue, pain, and global status. Strength of correlation was graded according to the recommendation of Cohen as follows: 1. Moderate correlation coefficient (.30 to .49) 2. Strong correlation coefficient (.50 to 1.0) [19].

## Results

### Population characteristics

The study sample consisted of 73 francophone patients, mean age 52 ± 9 years, 67(92%) female, and mean disease duration 12 ± 12 years. The 7 withdrawn patients did not differ demographically from the study sample. Demographic and disease related characteristics of the study sample are shown in Table 1, with scores on parameters of symptom and functional complaints comparable to other populations of patients with FM. The FSDC was easy to complete with an average completion time of 2 minutes, and the average scoring time was 1 minute.

**Table 1:** Sample characteristics for 73 FM patients

**Characteristics**	
Age, years	52 ± 9
Duration FM years	12 ± 12
Pain , VAS	6.0 ± 2.8
MPQ	40 ± 15
PDI	32 ± 17
Body Map	25 ± 12
Patient global severity, VAS	5.7 ± 2.5
FIQ	60 ± 21
HAQ	1.1 ± 0.78

LEGEND: FM: Fibromyalgia, VAS: Visual Analog Scale, MPQ: McGill Pain. Questionnaire, PDI: Pain Disability Index, FIQ: Fibromyalgia Impact Questionnaire, HAQ: Health Assessment Questionnaire.

### Correlation analysis

Test-retest reliability was between .600 and .888 for the 25 single items of the FSDC, and .912 for the total FSDC, with all correlations significant (p < 0.0001) (Table 2). Cronbach’s alpha was .846 for FSDC assessment 1, and .867 for FSDC assessment 2 indicating good internal consistency for both assessments.

**Table 2:** Paired samples correlations for the individual components of the FSDC in 73 FM patients

Fatigue	.667
Trouble thinking or remembering	.709
Waking up tired (unrefreshed)	.635
Pain or cramps in lower abdomen	.793
Depression	.781
Headache	.888
Shoulder L	.846
Shoulder R	.835
Hip L	.771
Hip R	.727
Upper arm L	.852
Upper arm R	.791
Lower arm L	.712
Lower arm R	.755
Upper leg L	.724
Upper leg R	.634
Lower leg L	.716
Lower leg R	.754
Jaw L	.830
Jaw R	.881
Chest	.891
Abdomen	.600
Lower back	.657
Upper back	.705
Neck	.723
Total /12	.733
Total /19	.922
Survey total /31	.912

### Validity analysis

In order to test construct validity the Spearman correlation coefficients between the total FSDC and study questionnaires were calculated. Construct validity showed significant correlations between the total FSDC and the total FIQ 0.670, HAQ 0.413, MPQ 0.562, global VAS 0.591 and pain VAS 0.663 (all p<0.001). The individual subcomponents of the FSDC including measurements for pain, fatigue, cognitive problems, awakening unrefreshed from sleep, and symptoms of abdominal pain, depression and headache were correlated with measurements of pain (pain VAS, MPQ, PDI, Body Map and FIQ pain), and fatigue, restfulness, and depression as measured by the FIQ (Table 3).

**Table 3:** Non-parametric Spearman’s ρ correlations between individual components of the FSDC and other measures

**Individual measures of FSDC**	**Pain-VAS**	**MPQ**	**PDI**	**Body map**	**FIQ- pain**	**FIQ-fatigue**	**FIQ-restfulness**	**FIQ-depression**
Pain	.660***	.562***	.644***	.793***	.604***	.538***	.445***	.240*
Fatigue	.414***	.214	.471***	.351**	.570***	.546***	.382**	.066
Cognitive problems	.347**	.307**	.398***	.461***	.487***	.355**	.348**	.080
Unrefreshing sleep	.380***	.355**	.465***	.241*	.557***	.563***	.423***	.166
Abdominal pain	.051	.060	.185	.025	.117	.065	-.084	-.213
Depression	-.112	.069	.010	-.178	-.123	-.055	.020	.390***
Headache	.220	.099	.265*	.254*	.259*	.187	.022	-.091

LEGEND: * p ≤ 0.05, **p ≤ 0.01, ***p ≤ 0.001.

## Discussion

In this study, we have demonstrated that the French version of the FSDC is a reliable instrument for measurement of symptom severity in patients with FM, with test-retest reliability coefficients ranging from .600 to .912, for both the individual component as well as total scores. We have also further demonstrated validity of this instrument with good correlation between the FSDC and other measures used to assess symptom complaint in FM. Therefore this study, conducted in a French speaking population of FM patients, further supports the usefulness of the FSDC in FM patients.

The FSDC was devised following the reevaluation of diagnostic criteria for FM, acknowledging that the symptomology of this syndrome extends beyond that of only body pain [10,11]. As FM presents a spectrum of severity rather than uniquely an all or none diagnosis, the composite and severity of symptoms, rather than solely a report of pain, should be addressed. Incorporating symptoms beyond pain will provide a more meaningful assessment of the global impact of this condition in an individual patient. Following the proposal for redefining diagnostic criteria, the working group has proposed a severity scale in order to grade severity of symptoms in a patient with FM [10]. The FSDC sets out to address these symptoms, with weighting equivalent to two thirds for pain, and one third for other symptoms. The maximum score for the FSDC is 31, with 13 suggested as a cutoff point to discriminate those with FM from those without FM [10]. The FSDC has been validated in English and in Japanese patients with FM, as well as patients with non-FM rheumatic disease [10,12]. This recognition of co associated symptoms with pain in FM is in line with increased neurophysiologic understanding of this condition [20].

When the new criteria for FM were proposed in 2010, the questionnaire devised for diagnostic purposes was originally completed both by the patient and physician [11]. In order to enable the questionnaire to be used for survey purposes and to introduce a severity scale, a fully patient-completed questionnaire, the FSDC, was developed [10]. This questionnaire measures the overall distress of FM and was previously termed the “fibromyalgianess scale” [10,21]. This composite of symptoms is however not unique to FM but may be applicable to patients with other chronic pain conditions. We therefore propose that the FSDC should now be renamed the “Polysymptomatic Distress Scale” (PDS). We believe that this clarification will simplify patient care. Further studies testing the validity of the FSDC now named the PDS in other rheumatic diseases and chronic pain conditions are required.

There are a number of points that require clarification. There is currently no single measurement for symptom severity in FM that is entirely comparable with the FSDC. The two measures that most closely align with the FSDC are the patient global assessment of disease severity and the FIQ [3]. The FIQ has a functional as well as a symptom component, but does not address symptoms of cognition or other somatic symptoms, whereas the FSDC measures only symptoms, and does not assess functional status. It is for this reason that we assessed the comparable individual components of pain, fatigue and sleep disturbance for the FIQ and FSDC in order to make direct comparisons. It is also acknowledged that the specific questions regarding pain, sleep disturbance, mood, and fatigue are nuanced differently in the FIQ and FSDC and could be open to different interpretations. However, even with these considerations, both the total FIQ and patient global assessment of disease severity, as well as individual measures of pain, fatigue, and unrefreshing sleep, correlated well with the FSDC indicating construct validity for assessment in patients with FM. An additional consideration is that all patients in our study had an established diagnosis of FM. It could be argued that these patients might express symptom severity at the extreme end of the spectrum and therefore would be more likely to show construct validity. Our patients are however similar to those reported by other authors [22].

We have also demonstrated validity for the pain component of the FSDC when compared to a number of other measures of pain assessment including a numerical count of pain areas marked on a body map, pain VAS, and MPQ. It is notable that the pain component of the FSDC, namely the WPI, assesses only the location of body pain, whereas the pain VAS assesses intensity, and the MPQ assesses mostly the emotional impact of pain. It is therefore interesting to note that the different pain measures correlated well with the WPI. This might suggest that the location or diffuseness of pain in FM may be associated with severity from the patient viewpoint.

An important consideration is the simplicity of the FSDC from the patient viewpoint, and the ease of scoring for the investigator. A questionnaire that requires simple numerical addition without need for any adjustments represents an attractive tool that might even become useful in clinical practice. The whole questionnaire is on a single page, allowing for a first impression as one that is less challenging than a lengthier document. Even though this questionnaire is remarkably simple and easy to administer in English, we followed a rigorous translation protocol and did not identify any important cultural adaptations that required attention. We do acknowledge the limitation of this study which was conducted at a single tertiary care centre and therefore recommend that further testing of the FSDC should be done at the primary care level.

## Conclusion

In conclusion, this new diagnostic and severity scale for FM performed well in the French translated version for patients with a diagnosis of FM. The FSDC, previously termed the “Fibromyalgianess Scale” which we propose should be renamed the “Polysymptomatic Distress Scale” (PDS), still requires further validation in other settings for patients with FM, as well as testing in other groups of patients with pain. Furthermore, the FSDC requires correlation with other meaningful and objective measures of function and disability in FM patients, such as sick-leave. If shown to be sensitive to change, this severity scale could be a useful tool for assessing outcome in both the clinical as well as the study setting. This questionnaire is unique in its simplicity, with completion by patients within a few minutes, and time to score measured as less than one minute. This tool has the potential to become the standard for measurement of symptom severity in FM and other chronic pain conditions.

## Competing interests

Dr. Fitzcharles has received consulting fees, speaking fees, and/or honoraria (less than $10,000 each) from Lilly, Pfizer, Purdue, and Valeant, and has given expert testimony for both the plaintiffs and defendants in medicolegal adjudication concerning pain in rheumatic conditions. Mr. Ste-Marie has no competing interests. Dr. Panopalis has received consulting fees, speaking fees, and/or honoraria (less than $10,000 each) from Abbott, Bristol-Myers Squibb, and Pfizer. Dr. Menard has no competing interests. Dr. Shir has received consulting fees, speaking fees, and/or honoraria (less than $10,000 each) from Astra-Zeneca, Janssen, Paladin, Pfitzer, and Purdue. Dr. Wolfe has no competing interests.

## Authors´ contributions

MAF designed the study. FW provided background on the validated measure. HM participated in the translation of the validated measure. PP lead the statistical analysis. PSM collected data. MAF, PSM, and YS wrote the manuscript. All authors read and approved the final manuscript.

## Pre-publication history

The pre-publication history for this paper can be accessed here:

http://www.biomedcentral.com/1471-2474/13/179/prepub

## Supplementary Material

Additional file 1 **Appendix.** Questionnaire sur l’échelle de diagnostic et de sévérité de la fibromyalgie. Click here for file
