# Supplementary material for: The 2010 American college of rheumatology fibromyalgia survey diagnostic criteria and symptom severity scale is a valid and reliable tool in a French speaking fibromyalgia cohort
Source: BMC Musculoskelet Disord. 2012 Sep 20;13:179. doi: 10.1186/1471-2474-13-179 (PMC3489797; doi:10.1186/1471-2474-13-179)
Supplement: Additional file 1 — Appendix. Questionnaire sur l’échelle de diagnostic et de sévérité de la fibromyalgie. [file 1471-2474-13-179-S1.doc]

**Questionnaire sur l’échelle de diagnostic et de sévérité de la fibromyalgie**

En utilisant l’échelle suivante, veuillez indiquer pour chaque item le degré de gravité des problèmes éprouvés **au cours de la dernière** **semaine** en cochant la case appropriée.

0 : Aucun problème

1 : Problèmes mineurs ou légers; habituellement légers ou intermittents

2 : Modérés; problèmes importants; survenant fréquemment et/ou à un niveau modéré

3 : Sévère: Problèmes continus qui ont un impact important sur la vie

Fatigue **🞏**0 **🞏**1 **🞏**2 **🞏**3

Problème de concentration ou de mémoire **🞏**0 **🞏**1 **🞏**2 **🞏**3

Se réveiller fatigué (sommeil peu réparateur) **🞏**0 **🞏**1 **🞏**2 **🞏**3

**Pendant les derniers 6 mois**, avez-vous eu les symptômes suivants ?

Douleur ou crampes intestinales : **🞏**Oui **🞏**Non

Dépression : **🞏**Oui **🞏**Non

Mal de tête : **🞏**Oui **🞏**Non

**DOULEUR ARTICULAIRE/CORPORELLE**

Veuillez indiquer si vous avez éprouvé de la douleur ou de la sensibilité **au cours des 7 derniers jours** dans les régions énumérées ci-dessous.

Veuillez marquer d’un X la case correspondante en prenant soin de préciser s’il s’agit du côté droit ou du côté gauche.

| **🞏**Épaule, gauche  **🞏**Épaule, droite | **🞏**Haut de la jambe, gauche  **🞏**Haut de la jambe, droit | **🞏**Bas du dos  **🞏**Haut du dos  **🞏**Cou |
| --- | --- | --- |
| **🞏**Hanche, gauche  **🞏**Hanche, droite | **🞏**Bas de la jambe, gauche  **🞏**Bas de la jambe, droit |
| **🞏**Bras supérieur, gauche  **🞏**Bras supérieur, droit | **🞏**Mâchoire, gauche  **🞏**Mâchoire, droite | **🞏**Aucune douleur dans ces  régions |
| **🞏**Avant-bras, gauche  **🞏**Avant-bras, droit | **🞏**Poitrine  **🞏**Abdomen |

16 février 2010
